# Physician burnout: can we make a difference together?

**DOI:** 10.1186/s13054-015-0990-x

**Published:** 2015-07-02

**Authors:** Matthew Siedsma, Lillian Emlet

**Affiliations:** Department of Critical Care Medicine, University of Pittsburgh Medical Center, 3471 Fifth Avenue, 1215 Kaufmann Building, Pittsburgh, PA 15213 USA

## Abstract

**Citation:**

West CP, Dyrbye LN, Rabatin JT, Call TG, Davidson JH, Multari A, Romanski SA, Hellyer JMH, Sloan JA, Shanafelt TF. Intervention to promote physician well-being, job satisfaction, and professionalism: a randomized clinical trial. JAMA Intern Med. 2014;174:527–33.

**Background:**

Despite the documented prevalence and clinical ramifications of physician distress, few rigorous studies have tested interventions to address the problem.

**Methods:**

*Objective:* To test the hypothesis that an intervention involving a facilitated physician small-group curriculum would result in improvement in well-being.

*Design:* A randomized clinical trial of practicing physicians. Additional data were collected on nontrial participants responding to annual surveys timed to coincide with the trial surveys.

*Setting:* Department of Medicine at the Mayo Clinic in Rochester, Minnesota between September 2010 and June 2012.

*Participants:* The study involved 74 practicing physicians in the Department of Medicine and 350 nontrial participants responding to annual surveys.

*Interventions:* The intervention involved 19 biweekly facilitated physician discussion groups incorporating elements of mindfulness, reflection, shared experience, and small-group learning for 9 months. Protected time (1 hour of paid time every other week) for participants was provided by the institution.

*Outcomes:* Meaning in work, empowerment and engagement in work, burnout, symptoms of depression, quality of life, and job satisfaction were assessed using validated metrics.

**Results:**

Empowerment and engagement at work increased by 5.3 points in the intervention arm vs. a 0.5-point decline in the control arm by 3 months after the study (*P* = .04), an improvement sustained at 12 months (+5.5 vs. +1.3 points; *P* = .03). Rates of high depersonalization at 3 months had decreased by 15.5 % in the intervention arm vs. a 0.8 % increase in the control arm (*P* = .004). This difference was also sustained at 12 months (9.6 % vs. 1.5 % decrease; *P* = .02). No statistically significant differences in stress, symptoms of depression, overall quality of life, or job satisfaction were seen. In additional comparisons including the nontrial physician cohort, the proportion of participants strongly agreeing that their work was meaningful increased 6.3 % in the study intervention arm but decreased 6.3 % in the study control arm and 13.4 % in the nonstudy cohort (*P* = .04). Rates of depersonalization, emotional exhaustion, and overall burnout decreased substantially in the trial intervention arm, decreased slightly in the trial control arm, and increased in the nontrial cohort (*P* = .03, *P* = .007, and *P* = .002 for each outcome, respectively).

**Conclusions:**

An intervention for physicians based on a facilitated small-group curriculum improved meaning and engagement in work and reduced depersonalization, with sustained results 12 months after the study.

## Commentary

Physician burnout is a growing epidemic with symptoms noted for one in three physicians [1]. In our own specialty, high levels of burnout have been found among both intensivists and nurses in several international studies [2, 3]. Burnout is characterized by three components: (1) emotional exhaustion or loss of passion for one’s work, (2) depersonalization or treating patients as objects, and (3) the sense that your work is no longer meaningful [4]. The body of medical literature on burnout has demonstrated significant professional repercussions including decreased patient satisfaction, increased medical errors and litigation, and the personal consequences of substance abuse and depression [5–7]. One proposed solution to physician burnout is to address physician wellness [8, 9]. Wellness can be defined as “the complex and multifaceted nature of physicians’ physical, mental, and emotional health and well-being” [8]. The limited amount of medical research in this area has focused on interventions at the level of the individual, such as enhanced resiliency or mindfulness [10–12]. This study by West et al. [13] was a clinical trial to assess the effectiveness of a multimodal organization-level intervention of best-evidence practices for treating burnout.

This study was a randomized, single-center, clinical trial designed to demonstrate whether a physician small group curriculum that met for 19 sessions (1 hour, biweekly for 9 months) would improve physician well-being compared with giving physicians 1 hour of protected time biweekly. In addition to the two study arms, survey data were collected from 350 nontrial physicians at both the baseline time period and 3 months post study completion. The two study arms completed eight different survey outcomes measures including the Maslach Burnout Inventory [4] and several other scales, 85–90 questions in total. The nontrial physicians completed an abbreviated survey with four questions. None of the measured outcomes showed a significant improvement in the intervention group during or at the end of the intervention phase of the trial. However, an intention-to-treat analysis indicated significant improvement in both engagement at work and rates of depersonalization in the intervention group at both 3 months (*P* = .04, *P* = .004) and 12 months (*P* = .03, *P* = .02) after the end of the study. None of the other six outcome measures significantly improved at any time they were measured. When the trial arms were compared with the nontrial cohort, the intervention group showed significant improvement in the areas of finding their work meaningful (*P* = .04) as well as lower rates of emotional exhaustion (*P* = .07), depersonalization (*P* = .03), and overall burnout (*P* = .002). There was no significant difference in quality-of-life measures between the two study arms and the nontrial cohort.

There are a number of strengths to the design of this trial. This study is the first randomized control trial (RCT) to test an organization-level intervention to reduce physician burnout. Previously, small group intervention trials were observational or descriptive in nature alone, such as Doctoring to Heal Balint groups [11]. Earlier RCTs focused on individual interventions such as mindfulness or resilience [12, 14]. The intervention was a well-planned curriculum divided into three modules of “self”, “patient”, and “balance” with each session following the same structure. The study is strengthened by the 12-month longitudinal follow-up to ensure there was a lasting effect from the intervention. Finally, the outcome measures were carefully selected, validated instruments for various domains including physician distress, well-being, and meaning in work.

There are several limitations to this study. West et al. carried out a single-center trial of only academic internal medicine physicians, and therefore the study cannot be directly generalized to other centers or physicians. There is concern whether the two arms of the trial and nonstudy cohorts are comparable at baseline. The data from Table one in West et al. [13] suggest that the physicians in the intervention arm already had a higher incidence of burnout prior to the start of the study compared with either the control arm or the nonstudy cohort. These data also suggest that physicians in both study arms suffered from higher incidence of work–home conflict compared with the nonstudy cohort. Taken together these results indicate that the physicians who agreed to participate in the study were already more distressed than their nontrial counterparts. The authors measured several different domains with eight validated instruments, and therefore it is difficult to quantify simple conclusions about the utility of the intervention. The results imply that any additional hour of protected nonclinical time confers a benefit because there was a greater difference between the nonstudy cohort and the trial groups than between the intervention and control groups.

Our primary conclusion is that there is no one panacea for all physicians suffering from burnout. The study does, however, suggest how we might improve physician well-being. Some physicians will benefit from an additional hour of protected nonclinical time for personal meditation while others will benefit from a shared community curriculum. Future research should focus on which groups of physicians will benefit from the different interventions described in previous studies.

## Recommendation

This study demonstrated improvement in two of the burnout domains (depersonalization, emotional exhaustion) and overall burnout after implementation of a physician wellness small-group curriculum. However there was no improvement seen in several other measures of physician well-being, and the physicians who were given an additional hour of protected time without the intervention showed similar improvement in physician distress domains. Given the ever increasing workload asked of physicians, consideration should be made at a practice management or institutional level on a breakpoint between faculty well-being and associated performance versus excess workload and increased burnout.

## Abbreviation

RCT: Randomized control trial
